# Health‐care interventions to promote and assist tobacco cessation: a review of efficacy, effectiveness and affordability for use in national guideline development

**DOI:** 10.1111/add.12998

**Published:** 2015-07-29

**Authors:** Robert West, Martin Raw, Ann McNeill, Lindsay Stead, Paul Aveyard, John Bitton, John Stapleton, Hayden McRobbie, Subhash Pokhrel, Adam Lester‐George, Ron Borland

**Affiliations:** ^1^ Cancer Research UK Health Behaviour Research CentreUniversity College LondonLondonUK; ^2^ Special Lecturer, UK Centre for Tobacco and Alcohol Studies, Division of Epidemiology and Public HealthUniversity of NottinghamNottinghamUK; ^3^ Professor of Tobacco Addiction, King's College London, UK Centre for Tobacco and Alcohol StudiesNational Addiction CentreLondonUK; ^4^ Cochrane Tobacco Addiction Group, Department of Primary Care Health SciencesUniversity of OxfordOxfordUK; ^5^ Professor of Behavioural Medicine, Nuffield Department of Primary Care Health Sciences, Radcliffe Observatory QuarterUniversity of OxfordOxfordUK; ^6^ Professor of Epidemiology, UK Centre for Tobacco and Alcohol Studies, Division of Epidemiology and Public HealthUniversity of NottinghamNottinghamUK; ^7^ Reader in Addiction Statistical Analysis, Addictions Department, Institute of PsychiatryKings College LondonLondonUK; ^8^ Reader in Public Health Interventions, Wolfson Institute of Preventive MedicineQueen Mary University of LondonLondonUK; ^9^ Health Economics Research GroupBrunel University LondonUxbridgeUK; ^10^ LeLan LtdBristolUK; ^11^ Cancer Council Victoria, Melbourne, VictoriaAustralia

**Keywords:** Affordability, behavioural support, brief interventions, cytisine, effectiveness, efficacy, interventions, NRT, smoking cessation, tobacco cessation

## Abstract

**Aims:**

This paper provides a concise review of the efficacy, effectiveness and affordability of health‐care interventions to promote and assist tobacco cessation, in order to inform national guideline development and assist countries in planning their provision of tobacco cessation support.

**Methods:**

Cochrane reviews of randomized controlled trials (RCTs) of major health‐care tobacco cessation interventions were used to derive efficacy estimates in terms of percentage‐point increases relative to comparison conditions in 6–12‐month continuous abstinence rates. This was combined with analysis and evidence from ‘real world’ studies to form a judgement on the probable effectiveness of each intervention in different settings. The affordability of each intervention was assessed for exemplar countries in each World Bank income category (low, lower middle, upper middle, high). Based on World Health Organization (WHO) criteria, an intervention was judged as affordable for a given income category if the estimated extra cost of saving a life‐year was less than or equal to the per‐capita gross domestic product for that category of country.

**Results:**

Brief advice from a health‐care worker given opportunistically to smokers attending health‐care services can promote smoking cessation, and is affordable for countries in all World Bank income categories (i.e. globally). Proactive telephone support, automated text messaging programmes and printed self‐help materials can assist smokers wanting help with a quit attempt and are affordable globally. Multi‐session, face‐to‐face behavioural support can increase quit success for cigarettes and smokeless tobacco and is affordable in middle‐ and high‐income countries. Nicotine replacement therapy, bupropion, nortriptyline, varenicline and cytisine can all aid quitting smoking when given with at least some behavioural support; of these, cytisine and nortriptyline are affordable globally.

**Conclusions:**

Brief advice from a health‐care worker, telephone helplines, automated text messaging, printed self‐help materials, cytisine and nortriptyline are globally affordable health‐care interventions to promote and assist smoking cessation. Evidence on smokeless tobacco cessation suggests that face‐to‐face behavioural support and varenicline can promote cessation.

## Introduction

Stopping smoking improves life expectancy and reduces the risk of chronic disease. The earlier the age of stopping, the greater the benefit; studies in the United Kingdom and United States estimate that stopping in young adulthood recovers an average of 10 years of life 1, 2. The health risks from other forms of tobacco use are mostly lower (in the case of Swedish‐type snus, much lower) than from smoking, but reducing prevalence of tobacco use in general is an important public health goal 3. Interventions that promote and assist smoking cessation are among the most cost‐effective life‐preserving interventions available in high‐income countries; interventions that can help as few as 1% of smokers to stop for at least 6 months can be highly cost‐effective ways of saving lives 4. Interventions to promote and assist smoking cessation include mass media campaigns, advertising bans, fiscal measures and legislation to ban smoking in indoor public areas 5, as well as ‘health‐care interventions’, i.e. interventions typically delivered or made available to individuals through a country's health‐care system 6.

Health‐care interventions to promote and support smoking cessation have a strong evidence base and are available in many countries. These interventions include brief advice and behavioural support from health‐care workers and a range of medications, including nicotine replacement therapy (NRT), varenicline, bupropion, nortriptyline and cytisine 7, 8, 9, 10. These interventions may be paid for directly by individual smokers, health insurance companies or the state. There is far less research on interventions to support cessation from other forms of tobacco use, but such evidence does exist.

The World Health Organization (WHO) Framework Convention on Tobacco Control (FCTC) 11 is a UN health treaty that sets out a range of measures that countries should take to reduce tobacco use. By October 2014 there were 179 Parties to the Treaty, making it one of the most widely adopted of all UN treaties. Article 14 of the Treaty obliges Parties to the treaty to ‘develop and disseminate… guidelines based on scientific evidence… and take effective measures to promote cessation… and treatment of tobacco dependence’. In November 2010 the fourth Conference of the Parties to the treaty adopted guidelines for the implementation of Article 14, which set out in more detail the steps Parties should take to develop tobacco cessation support systems 12. These guidelines recommend that Parties strengthen or create sustainable infrastructure to motivate quit attempts and ensure wide access to cessation support. However, a recent global survey shows that in many countries relatively little progress has been made on this 13.

This paper aims to assist the process of implementing Article 14 of the FCTC by assessing the efficacy, effectiveness and affordability of the major health‐care interventions to promote and assist tobacco cessation, in most cases relating to smoking 14. It provides a concise summary of the evidence which, with an accompanying affordability calculator spreadsheet, can be used in guideline development, and in selecting cessation interventions appropriate to a country's current situation and resources. Countries would otherwise need to undertake such a review themselves, a process that can be time‐consuming and costly, especially for low‐ and middle‐income countries 15.

We use Cochrane reviews of tobacco cessation interventions to obtain efficacy estimates in terms of the percentage of tobacco users in randomized controlled trials (RCTs) helped to stop using those interventions. We then review additional studies that may help to form a judgement about the real‐world effectiveness of the interventions, and contextual and implementation factors that may enhance or reduce this effectiveness. Then we estimate the incremental cost‐effectiveness ratios (ICERs) for the effective interventions for countries in different World Bank income categories. ICERs give the incremental cost incurred for an incremental health outcome, usually expressed as cost per life‐year gained or cost per quality‐adjusted life‐year gained. Because ICERs are the ratio of the change in costs to incremental benefits of an intervention, they are good measures to compare different interventions used in tobacco cessation and across countries. These ICERs can then be used to assess the affordability of the interventions globally taking account of income levels in relevant countries.

The paper and the affordability calculator have undergone extensive review, user testing and revision prior to submission as a published paper, with an advisory group of key stakeholders in low‐ and middle‐income countries, to help ensure that they meet their needs. The members of the advisory panel are listed in Box 1.

**Box 1. International advisory panel**

Lekan Ayo‐Yusuf, Director/Dean, Sefako Makgatho Health Sciences University, Medunsa, South Africa; Beatriz Champagne, Executive Director, InterAmerican Heart Foundation, Buenos Aires, Argentina; Elma Correa‐Acevedo, Pneumologist and Tobacco Clinic Coordinator, Instituto Nacional de Cancerología, México City, México; Thomas Glynn, Consulting Professor, Stanford Prevention Research Center, School of Medicine, Stanford University; USA; Feras Hawari, Director, Cancer Control Office and Chief, Section of Pulmonary and Critical Care, King Hussein Cancer Centre, Amman, Jordan; Hom Lal Shresha, Honorary Research Fellow, Non‐Smokers’ Rights Association of Nepal, Kathmandu, Nepal; Vimla Moodley, Director, Health Promotion, National Department of Health, South Africa; Caleb Otto, Ambassador and Permanent Representative of Palau to the United Nations, USA; Dennis Rada, Tobacco Control Coordinator, Inter‐American Heart Foundation, La Paz, Bolivia; Javier Saimovici, Chief of Home Care Section, Internal Medicine Department and Tobacco Control Program Member, Italian Hospital of Buenos Aires, Argentina; Oleg Salagay, Deputy Director, Department of International Cooperation and Public Relations, Ministry of Health of the Russian Federation, Moscow, Russia; Dan Xiao, Tobacco Medicine and Smoking Cessation Center in China‐Japan Friendship Hospital, Beijing, China

Considering ‘efficacy’ (the effect of an intervention in test conditions compared with a specified alternative) in more detail, Cochrane reviews provide rigorous, independent, quantitative estimates and are subject to a consistent quality control process. They provide meta‐analyses of RCTs usually with a minimum of 6‐month follow‐up, synthesizing data from direct head‐to‐head comparisons between interventions and comparison conditions. It is important to recognize that efficacy estimates based on the Cochrane reviews depend upon the particular circumstances of the trials conducted, including the control conditions used and the target population. However, they provide the best estimates available and a starting point for consideration of effectiveness.

There are many reasons why efficacy estimates from RCTs might not translate into the same level of effectiveness in practice 16, particularly when considering delivery in very different cultures and health‐care systems. We believe, therefore, that it is important to conduct an analysis of likely transferability to different contexts and to supplement efficacy estimates with evidence from ‘real‐world’ studies, which match as closely as possible the context in which the intervention would be delivered. Real‐world studies often have to be ‘observational’ (involving cohort studies, analysis of clinical records or surveys), rather than experimental. This is, in part, because willingness to be randomized to conditions in smoking cessation is a major source of sample bias. By themselves they cannot definitely establish a causal connection between interventions and outcomes. However, they can help to form a judgement about whether or not effect sizes found in RCTs are likely to translate into practice in the settings of interest.

An intervention may be effective but not affordable in many countries, particularly low‐ and middle‐income countries. This paper therefore uses the efficacy estimates, qualified if necessary by real‐world effectiveness evidence, to make affordability judgements. Affordability of treatments can be construed in a variety of ways 17, depending on whether the individual or the state is paying directly, and whether the treatment has to be used over a long period or can be used for a short duration.

We define as affordable for a given country an intervention for which the ICER was less than the per capita gross domestic product (GDP). This corresponds to the WHO definition of ‘highly cost effective’ 18. It is important to recognize that the ICERs for a given country rely upon estimates of background quit rates and other causes of mortality, as well as effectiveness of interventions, if they were to be delivered in that country. The background quit rates are mostly not known, and so we have to use data from countries where they are known and apply appropriate caveats to any conclusions drawn. In recognition of all these factors, we provide a model (available as a spreadsheet, with instructions, accompanying this paper) with which users can vary the parameters used for a given country to assess affordability across a range of user‐defined assumptions.

An important issue with regard to any tobacco cessation intervention is the question about how far it aims to promote attempts to quit versus helping quit attempts to succeed. This leads to what may be termed ‘the first law of tobacco cessation’, stated as:

E=N×S

where *E* is the number of ex‐tobacco users generated in a given period, *N* is the number of tobacco users attempting to stop and *S* is the probability of success of those quit attempts. Note that one may adopt different time‐periods for someone counting as an ex‐user, but for present purposes only permanent ex‐users are considered, except for smoking cessation in pregnancy.

While this equation represents an obvious truth, it serves to focus attention on the need to tailor intervention strategies to different contexts. For example, in populations where the rate of cessation attempts is low but success rates are high, it is more efficient to focus resources on interventions to boost *N*. In populations where *N* is already high and *S* is low, it may be more efficient to seek to boost *S*. Of course, *N* and *S* may affect each other. For example, availability of more effective methods of stopping may boost the rate of attempts to stop. It is necessary to consider these issues when considering the different interventions.

Related to this is the importance of considering the reach of an intervention. A 1 percentage‐point increase in cessation from intervention A that is delivered to 30% of smokers will have a greater population impact than a 5 percentage‐point increase from intervention B that is accessed by only 2% of smokers. Thus, some of the interventions being reviewed with low effectiveness may have a greater impact than others with high effectiveness.

This review covers all forms of tobacco use and is intended to be applicable globally. However, as noted earlier, almost all the evidence is from studies of smoking, and from high‐income countries. Where we have been able to find studies from low‐ and middle‐income countries we have included them. In the absence of evidence to the contrary we propose that evidence on smoking cessation should be applied to all forms of tobacco cessation, and in the absence of evidence from low‐ and middle‐income countries we propose that the evidence from high‐income countries be applied to all countries, interpreted in the light of national circumstances and priorities.

In summary, this paper provides a concise review of the evidence on tobacco cessation interventions for use in guideline development, and in selecting and prioritizing cessation interventions at the national level, and includes assessments of affordability using an approach based on the one used by the WHO. With such a diverse range of interventions and contexts, the conclusions must be stated in very broad terms and subject to major caveats. With this in mind, the review is accompanied by an affordability calculator (Supporting information) in which users can vary their estimates to assess the impact on affordability.

## Methods

### Efficacy estimation

The Cochrane Library was searched to identify all systematic reviews of the efficacy of health‐care tobacco cessation interventions. Health‐care interventions were defined as those that involved pharmacological treatment, advice or support from a health‐care worker, printed materials or automated systems delivered to individuals or groups. We have included the last two categories of intervention, because these are important and are often delivered by health‐care agencies. We have excluded mass media campaigns, although these can clearly promote cessation 19. We have also excluded electronic cigarettes and other electronic nicotine delivery systems because of the limited amount of available evidence in clinical settings and variability across such products 20. Evidence is accumulating rapidly on these, and it is likely that we will be able to include them in an update of this review in the next couple of years.

Major intervention types were identified from the reviews. Reviews relating to specific subgroups of smokers or specific components or forms of behavioural support (e.g. motivational interviewing, stage‐based interventions, use of incentives) were excluded in order to focus on broad intervention categories. Those intervention types with statistically significant overall efficacy relative to a comparator from meta‐analyses were selected for inclusion in this review. Supporting information, Appendix S1, shows the reviews used for primary effect size estimation. Where there was significant variation (‘heterogeneity’) in effect sizes from different studies, it was noted. Supporting information, Appendix S2, shows Cochrane reviews that were considered and not included or were used to qualify judgements about effect size.

The pooled effect sizes from the meta‐analyses were recalculated as the overall percentage‐point difference between intervention and comparator conditions together with 95% confidence intervals of the estimate, taking account of any heterogeneity in effect size across studies. Studies included in the Cochrane reviews typically involved 6–12 months of follow‐up, which allows confident extrapolation to permanent cessation in a way that can be used for cost‐effectiveness analyses 21. Another approach would have been to use the rate ratios (ratios of abstinence rates in intervention versus comparison conditions) from the published reviews and apply these to an assumed quit rate in the control condition. The results from this approach were very similar to those we obtained except in the case of cytisine, where applying the rate ratio to a common placebo quit rate resulted in higher effective size estimates than we have used.

In the case of some interventions the target population may be all smokers coming into contact with a clinical service, whereas in others it may be smokers who are willing to use a particular method to help them in a quit attempt. Evidence from high‐income countries suggests that, even where they are widely available, only about 5% of smokers currently use face‐to‐face behavioural interventions to help them stop, but more than 30% may use a medication such as nicotine replacement therapy, which they can buy from a shop or pharmacy 22, 23.

The key efficacy statements in this review take the following form:

- When given to [population category], [intervention category] has been found in multiple (≥ 2) RCTs to increase 6–12‐month abstinence rates by [range of values] compared with [comparator].

### Effectiveness judgements

For internationally applicable statements of effectiveness, it is important to recognize that the implementation of the intervention, the intervention provider and population of tobacco users may differ widely. For example, evidence on physician advice may or may not generalize to advice from other types of health‐care worker, and the effectiveness of a given type of health‐care worker may vary across cultures. Therefore, it is necessary to form judgements about probable transferability on the basis of inference and whatever relevant evidence is available.

A review was conducted using PubMed and Web of Science of studies of effectiveness of the interventions covered by the efficacy review. The initial search was conducted using labels to identify the intervention (e.g. ‘brief advice’, ‘counselling’, ‘behavioural support’, ‘telephone’, ‘nicotine replacement therapy’, ‘varenicline’, ‘bupropion’, ‘self‐help’, ‘internet’, ‘text messaging’) combined with ‘smoking cessation’ or ‘tobacco cessation’. Studies were included if they were RCTs that could provide information about the generalizability of the RCTs in the Cochrane reviews or where they were observational studies that estimated the incremental cessation rates in samples using the intervention, taking account of important confounding variables such as severity of dependence on tobacco 24. Judgements about real‐world effectiveness were made by consensus among the authors based on the efficacy findings supplemented by this additional information and analysis. The consensus process involved drafting propositions and going through an iterative process of re‐drafting until all the members of the authorship team and international advisory panel agreed.

### Affordability assessments

The starting points for affordability estimates were the efficacy estimates derived from the Cochrane reviews, qualified if necessary by effectiveness judgements. These were used to calculate incremental cost effectiveness ratios for life years gained (ICERs) based on cost information (see below). Because of the high variability in costs in different countries, particularly in the cost of health‐care worker time, and potentially different approaches to implementation, only broad estimates could be made. However, an affordability calculator is provided in Supporting information, File 2 to allow users to make estimates using their own data.

Our method of calculating ICERs 25 used life years gained rather than quality‐adjusted life years as the outcome. This is because there is insufficient information available to assess how far quality of life (QoL) may be affected in continuing smokers versus those who stop in different countries, and use of quality‐adjusted life years is subject to extensive debate 26, 27, 28. Stopping smoking improves health‐related quality of life at all ages, as well as life expectancy, and so it would be reasonable to expect quality‐adjusted life years gained to be at least as high as life years gained 29. However, this also depends upon how many of the additional life years are spent with poor quality of life, and this is subject to too much uncertainty for QoL to be taken into account in this review.

An effective intervention was judged to be ‘affordable’ for a given income category of country if the ICER was less than the per capita GDP of the reference country in that category (Table 2 and Supporting information). The countries chosen were Nepal, India, China and the United Kingdom (low‐, lower middle‐, upper middle‐ and high‐income, respectively), ones with good information on costs and close to the median per capita GDP for the income category. We considered an intervention that would be affordable in all four income categories to be globally affordable. The ICER threshold of one per capita GDP corresponds to the WHO definition of ‘highly cost effective’. We also used the model to assess affordability in a number of other countries representing all income categories and regions (Supporting information).

We used World Bank data for per capita GDP expressed in international dollars (http://data.worldbank.org/indicator/NY.GDP.PCAP.PP.CD), the WHO table of costs of health‐care providers for the different WHO regions for staff costs (http://www.who.int/choice/costs/prog_costs/en/index13.html) and expert sources for local costs of medications for set‐up and ancillary costs.

Medication costs may vary over time, with particular arrangements with manufacturers and to some degree in different countries. For our model we took an approximate average price estimate based on the information from countries where data were available. Where prices differ, this can be reflected in user input to the model. We recommend re‐computing affordability estimates when estimates of drug costs differ from those assumed here. This can occur when drugs come out of patent and generic manufacturers enter the market, or where governments purchase products at discounted rates.

Where materials and service costs are involved (e.g. automated text messaging), we have used an upper estimate and incorporated the set‐up costs into a unit cost per quit attempt assuming a minimum number of users. Full details of assumptions underlying costs are given in the spreadsheet (Supporting information) notes. Our general principle has been to estimate costs at the upper end of a plausible range so that affordability assessments will be conservative.

Note that our affordability assessment takes no account of savings in societal, individual or health‐care costs resulting from stopping tobacco use. In many countries, these are considerable in the case of smoking and mean that many cessation interventions produce a net financial benefit over a given time‐span.

## Results

The efficacy, effectiveness and affordability of the major categories of intervention are presented in this section, with the behavioural interventions considered first and then the pharmacological interventions. Within each of these categories, we consider the interventions in approximate order in which effectiveness was first established by Cochrane reviews. Table 1 summarizes the main conclusions concerning efficacy while Table 2 shows assessments of affordability.

**Table 1:** Efficacy of health‐care smoking cessation interventions from Cochrane reviews.

Intervention versus comparison	Delivered by	Delivered to	Percentage point increase in 6–12‐month abstinence (95% CI)	Projected percentage point increase in 6–12‐month abstinence compared with no intervention
Brief advice from a physician versus no intervention	Physicians	Smokers attending a surgery	2 (2–3)	2
Printed self‐help materials versus nothing	Health‐care provider (e.g. health promotion organization)	Smokers wanting help with stopping and willing to set a quit date	2 (1–3)^a^	2
Proactive telephone support versus reactive telephone support	Trained stop‐smoking practitioners	Smokers wanting help with stopping and willing to set a quit date	3 (2–4)^a^, ^b^	5
Automated text messaging versus non‐smoking‐related messaging	Systems providers	Smokers wanting help with stopping and willing to set a quit date	4 (3–5)^a^	4
Face‐to‐face individual behavioural support versus brief advice or written materials	Trained stop‐smoking practitioners	Smokers wanting help with stopping and willing to set a quit date	4 (3–5)^b^	6
Face‐to‐face group‐based behavioural support versus brief advice or written materials	Trained stop‐smoking practitioners	Smokers wanting help with stopping and willing to set a quit date	5 (4–7)^b^	7
Single NRT versus placebo	Health professionals^c^	Smokers wanting help with stopping and willing to set a quit date	6 (6–7)^d^	6
Dual form/combination NRT versus placebo	Health professionals^c^	Smokers wanting help with stopping and willing to set a quit date	11^e^	11
Cytisine versus placebo	Health professionals	Smokers wanting help with stopping and willing to set a quit date	6 (4–9)^f^	6
Bupropion versus placebo	Health professionals	Smokers wanting help with stopping and willing to set a quit date	7 (6–9)^f^	7
Nortriptyline versus placebo	Health professionals	Smokers wanting help with stopping and willing to set a quit date	10 (6–15)^f^	10
Varenicline versus placebo	Health professionals	Smokers wanting help with stopping and willing to set a quit date	15 (13–17)^f^	15

a Significant heterogeneity.

b Use of an active control may mean that the total effect size versus nothing is larger.

c Health‐care worker qualified to prescribe or provide the medication.

d No clear differences between products or interaction with intensity of behavioural support, but some evidence that higher‐dose products are more effective than lower‐dose ones.

e Synthetic estimate based on incremental effect of dual‐form nicotine replacement therapy (NRT) compared with single‐form.

f Studies were undertaken in the context of multi‐session face‐to‐face behavioural support. CI = confidence interval.

**Table 2:** Affordability^a^ of health‐care smoking cessation interventions.

**Intervention** b	**Affordability**			
	Low‐income (Nepal)	Lower‐middle‐income (India)	Upper‐middle‐income (China)	High‐income (UK)
**Automated text messaging**	**7.7**	**11.2**	**25.9**	**109.5**
**Brief health‐worker advice**	**2.7**	**7.8**	**18.0**	**12.3**
**Printed self‐help materials**	**2.4**	**4.6**	**10.8**	**19.3**
**Cytisine**	**1.7**	**4.9**	**11.3**	**15.0**
**Nortriptyline**	**1.4**	**4.1**	**9.5**	**8.6**
**Proactive telephone support**	**1.0**	**3.8**	**9.7**	**4.5**
Face‐to‐face behavioural support^c^	0.9	**3.4**	**8.6**	**4.0**
Bupropion	0.5	**1.6**	**3.7**	**7.7**
Varenicline	0.5	**1.3**	**3.0**	**9.2**
NRT (single)^d^	0.4	**1.0**	**2.4**	**6.9**

a Affordability is the ratio of per capita gross domestic product (GDP) to the cost per life year gained, i.e. in order for an intervention to be affordable, the ‘additional’ cost of saving a life‐year must be equal to or less than a country's per capita GDP (WHO criteria for ‘highly cost‐effective’); e.g. an affordability score of 2 means that the ‘extra’ costs required to save each life year is half of a country's per capita GDP (hence the intervention in question is affordable).

b Affordable interventions are marked in bold type.

c Only individual support is included.

d Dual‐form/combination nicotine replacement therapy (NRT) (transdermal patch plus a faster‐acting form) is more effective than single‐form, but assessing effectiveness and affordability relative to no pharmacotherapy would require indirect comparisons and so are not included here.

With regard to efficacy statements, some interventions use active comparators as the control condition. For example, face‐to‐face support usually uses brief advice as a comparator which would, in itself, be expected to have an effect. This means that the total effect of some interventions will be underestimated. Therefore, Table 1 also shows a projected estimate of the total effect size of the interventions. To be conservative, the affordability estimates use the lower figure, but users can enter the total effect size estimate in the affordability calculator if they wish.

Some interventions would be expected to combine in terms of effectiveness. For example, combining medication to behavioural support is expected to produce an approximately additive effect.

### Behavioural interventions

#### Brief advice

Brief opportunistic advice involves a health‐care worker raising the topic of smoking with a patient, advising the patient to stop and/or offering support and follow‐up. It would normally be expected to take no more than 20 minutes and most of the interventions evaluated took considerably less time than this (usually approximately 5 minutes).

##### Efficacy

When given by a physician to unselected smokers attending a consultation for a medical condition, brief smoking cessation advice has been found in multiple RCTs to increase 6–12‐month continuous abstinence rates by an average of 2 percentage points [95% confidence interval (CI) = 2–3] compared with doing nothing or usual care, with an advantage for more intensive compared with minimal advice (> 20 minutes) (see Table 1) 30. Evidence for efficacy of brief opportunistic advice to stop by other health‐care workers is suggestive rather than conclusive 31, 32.

There is insufficient evidence to draw conclusions regarding the efficacy of brief opportunistic health‐care worker advice to promote cessation of use of tobacco products other than cigarettes.

##### Effectiveness

Most of the RCTs thus far have been in high‐income countries with health‐care systems that have near‐universal coverage, and relates only to physician advice. There is no reason to believe that health‐care worker advice would be less effective in low‐ and middle‐income countries. It may be more effective in populations with minimal or no history of quitting, because the main effect is in prompting quit attempts. A recent RCT involving non‐medical health‐care workers delivering brief advice on tobacco use cessation door‐to‐door in slum areas of Delhi found an increase in 6‐month biochemical verified continuous abstinence rates of 2 percentage points (95% CI = 0.2–3) 33. Another RCT evaluated a brief motivational intervention delivered by health‐care workers in tuberculosis (TB) clinics in South Africa and found a doubling of the smoking cessation rates 34. Unfortunately, tobacco use is very high in health‐care workers in many countries 35, and this could reduce the effectiveness of advice from such workers. The FCTC Article 14 guidelines stress that this issue needs addressing, with help for health‐care workers to stop 11.

Countries vary considerably in how their health‐care systems are organized, including who would deliver brief advice and how they would be trained and motivated to give brief advice routinely. Nevertheless, brief advice is potentially usable globally with a very wide reach.

Our analysis is that brief advice to stop smoking and offer of support from a health‐care worker can have a small but important effect in promoting smoking cessation in any health‐care system that invests in the necessary training and support for this activity.

##### Affordability

With an effect size of 2 percentage points at 6–12 months, brief advice involving an average of 20 minutes of health professional time is globally affordable (see Table 2 and Supporting information). Given that many of the studies of brief advice have involved much less than 20 minutes of advice and that we have based the costs on physician time, the affordability is likely to be greater than we have estimated.

#### Face‐to‐face behavioural support

Behavioural support involves advice, discussion and encouragement, and other activities designed to (1) maximize motivation to remain abstinent, (2) minimize motivation to smoke, (3) enhance the skills and capacity needed to avoid and resist urges to smoke and (4) optimize effective use of stop‐smoking medication where available 36, 37. It can be delivered individually or in groups. The studies conducted to date have usually involved multiple sessions provided by specially trained health professionals over a period from 1 to more than 4 weeks following a target quit date.

##### Efficacy

When given to smokers who set a quit date and who are willing to receive such help, individual face‐to‐face behavioural support has been found in multiple RCTs to increase 6–12‐month continuous abstinence rates by 4 percentage points (95% CI = 2–5) compared with provision of written materials or brief advice 38. Group support has been found to increase 6–12‐month continuous abstinence rates by 5 percentage points (95% CI = 4–7) compared with printed self‐help materials 39. There is insufficient evidence from RCTs to draw conclusions about whether group‐based support is more effective than individual support 39. Behavioural support has been found to add to the efficacy of medication 40. Behavioural support has also been found to be effective in helping smokeless tobacco users to quit 3.

##### Effectiveness

Evidence from the English stop‐smoking services, which currently treat approximately more than 500, 000 smokers each year, shows success rates lower than would be expected from the RCT evidence but greater than would be expected from brief advice. There is considerable variability between different local services and individual practitioners 41, 42. This highlights the importance of careful staff selection, training and assessment as well as close monitoring of outcomes 43.

In the English services, group support appears to be more effective than individual support, and specialist practitioners (those trained and employed full‐time specifically to help tobacco users stop) appear to be more effective than those delivering it occasionally in addition to other clinical duties 42.

Randomized trials in Pakistan and Malaysia have found behavioural support delivered as part of a TB screening and treatment service to have an effect on tobacco cessation 44, 45, suggesting strongly that this kind of intervention is practicable and effective in middle‐income countries when integrated into existing care pathways. Several studies in Arabic‐speaking populations have been carried out, with mixed results 46. However, the quality of the studies is low, limiting the conclusions that can be drawn.

Overall, there is reason to believe that face‐to‐face behavioural support delivered by trained health‐care workers with content developed from that used in RCTs would be effective in any health‐care system. There are, however, likely to be significant practical and financial barriers to widespread implementation of programmes of this kind in many countries, except where they can be incorporated into existing health‐care provision.

##### Affordability

With an effect size of 4 percentage points at 6–12 months, face‐to‐face behavioural support involving an average of 180 minutes of health‐worker time per quit attempt would be affordable in most middle‐ and high‐income countries, but not in low‐income countries (Table 2 and Supporting information).

#### Printed self‐help materials

Printed self‐help materials include leaflets, booklets and books designed to provide encouragement, advice and support to maximize motivation to stop smoking, reduce motivation to smoke, enhance self‐regulatory skills and capacity and, in some cases, to optimize medication use.

##### Efficacy

When given to smokers wanting help with a quit attempt, printed self‐help materials have been found in multiple RCTs to increase quit rates by 2 percentage points (95% CI = 1–3) compared with no intervention 47. There was significant heterogeneity in the effect sizes.

##### Effectiveness

It seems reasonable to assume that self‐help materials would be effective in a wide range of contexts and populations. It is likely that literacy would be a significant factor limiting use, given that none of the materials evaluated relied exclusively on images. The content and style of the materials would be expected to play an important role in effectiveness, but there is no evidence to indicate what specific components should be included.

Overall, printed self‐help materials would be expected to be useful in helping smokers to stop in a wide range of settings.

##### Affordability

If self‐help materials could achieve an effect size of 2 percentage points at 6–12 months, they would be globally affordable (see Table 2 and Supporting information). If they can be distributed electronically via the internet, they become even cheaper (as long as internet access is widespread and inexpensive), as there are no printing and distribution costs. It is also possible that in some countries many smokers would buy self‐help books or booklets themselves, so that there would be no cost to state funders.

#### Telephone support (quitlines)

Telephone support involves similar broad categories of activity to face‐to‐face support (see above). It can be ‘proactive’ or ‘reactive’. In proactive support a trained counsellor initiates calls, following an initial enquiry by the caller, to provide support according to an agreed schedule, while in a reactive model support is available on demand to people who call a quitline number.

##### Efficacy

When given to smokers wanting help with stopping, proactive telephone support has been found in multiple RCTs to increase 6–12‐month continuous abstinence rates by 3 percentage points (95% CI = 2–5) compared with the offer of reactive telephone support only 48. Significant heterogeneity exists in the effect sizes of different RCTs, with one large study showing no effect 49. This means that we know that telephone support can be effective with the right content, delivery and in the right context, but such factors can make a substantial difference. The effect of proactive telephone support has been found both for smokers seeking help and those asked if they wanted support 50. There is insufficient evidence from RCTs to draw conclusions about the efficacy of reactive telephone support. However, as it often includes either or both provision of self‐help materials and/or brief advice from a health professional, it is likely to be similarly effective to them.

##### Effectiveness

A large pragmatic RCT of multi‐lingual proactive telephone support versus self‐help materials in Asian smokers in California found a clear benefit 51. A recent study in England failed to show benefit from adding additional calls and the offer of free NRT to the standard proactive service, although NRT is readily available to buy or on prescription in that country and there was little difference in usage between intervention control conditions 52.

Our analysis is that there is good reason to believe that proactive telephone support can aid smoking cessation in any country, although it would be important to monitor success rates to establish that a specific service was delivering expected results.

##### Affordability

With an effect size of 3 percentage points at 6–12 months over reactive support and an average of 120 minutes of contact time per quit attempt, telephone support would be globally affordable (see Table 2 and Supporting information).

#### Automated text messaging

Automated text messaging aims to deliver content similar to face‐to‐face behavioural support, focusing on motivational messages, advice on coping with cravings and providing behavioural distraction when needed 53.

##### Efficacy

When given to smokers wanting help with stopping, automated text messaging interventions have been found in multiple RCTs to increase 6–12‐month continuous abstinence rates by 4 percentage points (95% CI = 3–5) compared with text messaging programmes providing generic health advice 54. There is significant heterogeneity in the effect sizes of the studies.

##### Effectiveness

As long as the content of programmes on offer to smokers is based on what was in the successful RCTs, there is reason to believe that broadly similar effects to those found in the RCTs would be seen. A large trial of a text messaging intervention, Text2Stop, showed a clear benefit 55, and an analysis of the content of this intervention has been published 56 which could form a basis for development of other programmes.

Our analysis is that automated text messaging programmes can support smokers to stop and the content of the programmes should be based on those that have shown a clear benefit.

##### Affordability

With an effect size of 4 percentage points at 6–12 months, automated text messaging is globally affordable (see Table 2 and Supporting information), and could potentially have very good reach, as some low‐ and middle‐income countries have high mobile phone ownership. Affordability will vary for governments as a function of who bears the phone costs, the government or the individual, but even if text messaging were entirely government‐funded it is still likely to be one of the most affordable of all interventions.

#### Pharmacological interventions

#### NRT

NRT consists of products designed to deliver nicotine into the body in a form that does not involve smoking or ingestion of other toxins. The forms currently licensed for use in at least some countries of the world are 16‐ or 24‐hour transdermal patches, 2‐ or 4‐mg chewing gum, 1‐, 1.5‐, 2‐ or 4‐mg nicotine lozenges, 2‐mg sublingual tablet, nasal spray, inhalator, buccal pouch and mouth spray. Other nicotine products (e.g. some types of electronic nicotine delivery devices) are likely to be added to this list of licensed medicines in the coming years.

Smokers typically use these products starting on the designed target quit day and continuing for up to 12 weeks. Use can be started before the quit date, and they can be used for smoking reduction with a view to quitting at a later date.

##### Efficacy

When given to smokers of 15 or more cigarettes per day who are making a quit attempt on a pre‐specified day and willing to use NRT, multiple RCTs have shown that this type of aid increases 6–12‐month continuous abstinence rates by 6 percentage points (95% CI = 6–7) compared with placebo 57. These studies all involved some degree of contact with a health professional. There is no evidence from RCTs that the amount of health professional contact makes a difference to the effectiveness of the NRT although, as noted above, behavioural support has been shown to have an additive effect in and of itself 40.

There is insufficient information to draw conclusions about whether one form of NRT is likely to be more effective than another, overall, but evidence suggests that higher‐dose forms may be more effective than lower‐dose forms 57.

Evidence from multiple RCTs has shown that combining a nicotine patch with a faster‐delivery form of NRT (such as gum) increases 6–12‐month abstinence rates by 5 percentage points compared with single‐form NRT (95% CI = 3–7) 57.

NRT has not been adequately evaluated in smokers of fewer than 10 cigarettes per day and most studies have involved smokers of 15 or more cigarettes per day.

There is insufficient evidence from RCTs to draw firm conclusions as to whether or not NRT is an effective aid for smokeless tobacco cessation 58.

##### Effectiveness

Several cross‐sectional surveys have been reported as having failed to find that smokers using NRT to aid a quit attempt are more likely to still be abstinent at the time of the survey 59, 60, 61, but the data have, in some cases, been misinterpreted. Importantly, they failed to adjust adequately for major confounding variables such as dependence, and it is known that more dependent smokers opt to use NRT 62, 63. A large cross‐sectional study and a prospective study that adjusted for nicotine dependence found an effect of NRT when prescribed or given by a health professional that was similar to that found in RCTs, but no effect when NRT was bought over the counter 64, 65. Two other adequately designed prospective studies with sufficient power to detect an effect of NRT found that, adjusting for major confounding factors, smokers who used NRT in their most recent quit attempt were more likely than those who did not to maintain abstinence 66, 67. Both were multi‐national cohort studies. One involved follow‐up for 6 months and the other for a year; the effect size in both cases was in line with what would be predicted from the RCTs. It has also been shown that smokers in the English stop‐smoking services who use NRT are more likely to succeed in the short term than those who elect not to use any medication 41. A note of caution is sounded by the fact that the only RCT of nicotine patches in a low‐income country failed to find an effect 68.

Overall, our analysis suggests that NRT in the context of at least some behavioural support can aid smoking cessation in moderately heavy or heavy smokers. It appears not to be effective when bought from shops with no behavioural support. Combining transdermal patches with a faster‐acting product such as chewing gum or lozenge is more effective than using either alone.

##### Affordability

With an effect size of 6 percentage points at 6–12 months, and assuming up to 40 minutes of health‐worker time to explain and supervise use, NRT is affordable in middle‐ and high‐income countries but not in low‐income countries (see Table 2 and Supporting information). Generic NRT can now be produced cheaply, and this will increase its affordability. Prescribing combination NRT (patch plus a faster‐acting form) is likely to be more cost‐effective because of the additional effectiveness of the medication without an increase in prescriber time for supervision (see Supporting information).

#### Bupropion/amfebutamone (sustained release)

Bupropion hydrochloride is an atypical antidepressant that has multiple actions in the brain involving dopamine and noradrenaline pathways and may also act as a nicotinic antagonist. A typical course is 300 mg per day for 7–8 weeks, beginning a week prior to the designated quit date.

##### Efficacy

When used by smokers of 15 or more cigarettes per day, multiple RCTs have shown that this medication increases 6–12‐month continuous abstinence rates by 7 percentage points (95% CI = 6–9) compared with placebo 69.

There is insufficient evidence from RCTs to draw conclusions about whether 300 mg (the standard dose) is more effective than 150 mg 69.

Extending the course of treatment beyond 8 weeks appears to increase abstinence rates while the medication is being taken, but not when assessed 12 months after the medication has been discontinued 69.

Bupropion has not been shown to be effective for treating users of smokeless tobacco 3.

Evidence suggests that bupropion is broadly similar in effectiveness to NRT 69.

##### Effectiveness

Bupropion has not been tested without behavioural support. Smokers who use it in the English stop‐smoking services have similar short‐term success rates to those who use single‐form NRT and higher than those who use no medication 41. An RCT undertaken in English clinical services found bupropion and the combination of bupropion and NRT to produce similar 6‐month continuous abstinence rates to single‐form NRT 70. A prospective multi‐national population‐level study also found that, after adjusting for potential confounding variables, smokers who used bupropion in a quit attempt were more likely than those who did not to succeed in stopping 67. A note of caution is sounded by the failure of the only RCT of bupropion in a non‐high‐income country to show a benefit, although treatment groups were not equivalent and smokers had suspected tuberculosis, so further trials are needed 44.

Our analysis is that bupropion is a useful aid to smoking cessation in moderately heavy or heavy smokers, at least in the context of behavioural support, and is at least as effective as single‐form NRT.

##### Affordability

With an effect size of 7 percentage points at 6–12 months, bupropion, together with approximately 60 minutes of health professional time for screening and checking of adverse reactions, is affordable in middle‐ and high‐income countries but not in low‐income countries (see Table 2 and Supporting information). One hour of physician time has been specified for supporting medication use because the safety profile and contraindications of bupropion mean that a prescriber would need to spend some time addressing these.

#### Nortriptyline

Nortriptyline is a tricyclic antidepressant. For smoking cessation the dose is typically 75–100 mg per day for 12–14 weeks, starting 1 week before the quit date. Because of the side‐effect profile, it needs close supervision to monitor and possibly adjust the dose.

##### Efficacy

When used by smokers of 15 or more cigarettes per day nortriptyline has been found in multiple RCTs to increase 6‐month continuous abstinence rates by 10 (95% CI = 6–15) percentage points compared with placebo 69. Minor adverse events are common, particularly dry mouth, but these rarely cause discontinuation of treatment.

##### Effectiveness

Nortriptyline has not been evaluated without behavioural support. There is a reasonable presumption that similar tricyclic antidepressants such as amitriptyline are also effective, but these have not been tested directly.

A randomized trial among prisoners in Australia did not find a benefit from nortriptyline over and above behavioural support 71. An observational study in Brazil found smokers who used nortriptyline achieved abstinence rates at least as high as those using bupropion or NRT 72. A randomized trial in the United Kingdom found no benefit to adding nortriptyline to NRT 73.

Our assessment is that nortriptyline in moderately heavy or heavy smokers in the context of behavioural support aids smoking cessation. Patients using it experience some minor adverse reactions, particularly dry mouth, but these are not sufficient to undermine effectiveness.

##### Affordability

Nortriptyline is inexpensive, although the side‐effect profile of the drug requires more extensive supervision. With an effect size of 10 percentage points at 6–12 months, assuming 120 minutes of prescriber time for supervision, nortriptyline is affordable globally (see Table 2 and Supporting information).

#### Varenicline

Varenicline is a partial agonist designed to bind with high affinity to the nicotinic acetylcholine receptor composed of alpha‐4 beta‐2 subunits 74. A standard course of treatment is 1 mg per day beginning 1 week before the designated quit 74, then 11 weeks at 2 mg per day. Minor side effects reported are nausea and sleep disturbance. These do not appear to lead to significant treatment discontinuation. Reports during post‐marketing surveillance of raised risk of serious neuropsychiatric and cardiac adverse events have not been confirmed by controlled studies 75, 76, 77, 78.

##### Efficacy

When used by smokers of 15 or more cigarettes per day, multiple RCTs have shown that this medication increases 6–12‐month continuous or sustained abstinence rates by 15 percentage points (95% CI = 13–17) compared with placebo and 7 percentage points (95% CI = 4–11) compared with bupropion 74.

Evidence from RCTs suggests that varenicline is more effective than nicotine patches 74.

One trial has shown varenicline to help people stop using smokeless tobacco 3.

##### Effectiveness

Varenicline has not been evaluated in RCTs without what would be considered intensive behavioural support (multiple sessions with at least 120 minutes of total contact time), but an international cohort study strongly suggested effectiveness in routine clinical practice 67. Evidence from the English stop‐smoking services supports findings from RCTs that varenicline is more effective than bupropion or single‐form NRT 41.

Our assessment is that varenicline in moderately heavy or heavy smokers in the context of behavioural support aids smoking cessation. On average, it is more effective than bupropion and single‐form NRT and at least as effective as combination NRT. Although there is little reason at present to believe that it can cause serious side effects, concerns have been raised and it has been suggested that health‐care workers show particular vigilance in case these emerge.

##### Affordability

With an effect size of 15 percentage points at 6–12 months varenicline, together with approximately 60 minutes of health professional time, is affordable in middle‐ and high‐income countries, but not in low‐income countries (see Table 2 and Supporting information).

#### Cytisine

Cytisine is a partial agonist binding with high affinity to the nicotinic acetylcholine receptor composed of alpha‐4 beta‐2 subunits 74. A standard course of treatment is 4 weeks, beginning 1 week before the designated quit date, with a dosing regimen that reduces over time. It was the first medication ever to be licensed as a smoking cessation aid, and has been in use in eastern Europe for more than 40 years. In Russia and Poland it is available for purchase over the counter. No serious side effects have been detected. Nausea is a common minor side effect but does not lead to significant discontinuation of treatment.

##### Efficacy

When used by smokers of 15 or more cigarettes per day, cytisine has been found in multiple RCTs to increase 6–12‐month continuous abstinence rates by 6 percentage points (95% CI = 4–9) compared with placebo 74.

##### Effectiveness

An observational study from a large clinic in Warsaw found 12‐month biochemically verified abstinence rates somewhat greater than was found in a large clinical trial in the same clinic 79, 80. This suggests that the results of the RCTs would translate into at least as high success rates in routine clinical practice.

An open‐label RCT in smokers calling the New Zealand telephone helpline found cytisine to be more effective than nicotine replacement therapy 81.

There are more than 4 million users on the European Medicines Agency drug safety database, and no evidence has emerged of any safety problems, suggesting that this drug has a benign safety profile and is suitable for purchase with minimal or no medical supervision, as is currently the case in Russia and Poland.

Our assessment is that cytisine is an effective aid to cessation in moderately heavy to heavy smokers.

##### Affordability

With an effect size of 6 percentage points at 6–12 months, and requiring relatively little clinical supervision (approximately 40 minutes), cytisine would be globally affordable (see Table 2 and Supporting information).

## Discussion

Table 3 gives a narrative summary of the judged effectiveness and affordability of the interventions reviewed. All are judged to be affordable in middle‐ and high‐income countries and many are affordable in low‐income countries.

**Table 3:** Narrative summary of main conclusions.

Intervention	Effectiveness	Affordability
Brief opportunistic advice from a health‐care worker	This is an effective means of promoting tobacco cessation. The main issue is likely to be motivating and training health workers to deliver this intervention routinely as well as ensuring that the health‐care worker is not a tobacco user. It may be that offering help with stopping to all tobacco users provides optimum results	Globally affordable
Printed self‐help materials	This is an effective means of promoting tobacco cessation. It will be important to match intervention content as closely as possible to what has been found to be effective	Globally affordable
Proactive telephone support	This is an effective means of promoting tobacco cessation. Effectiveness will depend upon having in place appropriate procedures for selection, training, assessment and professional development of practitioners as well as evidence‐based treatment protocols	Globally affordable
Automated text messaging	This is an effective means of promoting tobacco cessation. It will be important to match intervention content as closely as possible to what has been found to be effective	Globally affordable
Face‐to‐face behavioural support	This is an effective means of promoting tobacco cessation. In many countries it may need to be integrated into existing services (e.g. tuberculosis screening). Effectiveness will depend upon having in place appropriate procedures for selection, training, assessment and professional development of practitioners as well as evidence‐based treatment protocols. Its effect appears to be broadly additive to medication if that is being used	Affordable in middle‐ and high‐income countries
Nicotine replacement therapy	This is an effective intervention when provided by a health‐care worker. Best results can be achieved by combining a transdermal patch with a faster‐acting form	Affordable in middle‐ and high‐income countries
Cytisine	This is an effective intervention when provided by a health‐care worker	Globally affordable
Bupropion	This is an effective intervention when provided by a health‐care worker. It is broadly similar in effectiveness to single‐form NRT	Affordable in middle‐ and high‐income countries
Nortriptyline	This is an effective intervention when provided by a health‐care worker	Globally affordable
Varenicline	This is an effective intervention when provided by a health‐care worker. It is more effective than bupropion and single‐form NRT	Affordable in middle‐ and high‐income countries

NRT = nicotine replacement therapy.

The most effective combination of interventions on the basis of our review is face‐to‐face behavioural support together with combination NRT or varenicline. However, this combination is currently only likely to be affordable in middle‐ and high‐income countries (although, of course, users who can afford it may choose to pay for it).

A major outcome of this review is that, based on reasonable assumptions about cost, and not taking any account of cost savings from tobacco cessation, health‐care systems of countries in every World Bank income category should be able to afford to implement smoking cessation interventions of established efficacy. Most notably, these are brief advice from a health‐care worker, proactive telephone support, printed self‐help materials, text messaging support and provision of medication such as cytisine or nortriptyline. Clearly, there are major practical challenges to achieving this and countries not currently doing this will need to give careful consideration to the pace of development that can be achieved, and which interventions to prioritize. Nevertheless, this review and our affordability calculator offer a basis for countries to establish what level of cessation provision they might aim for.

It is important to note that the affordability judgements are very broad, and do not take into account important factors that may be operating in particular countries. For example, in countries such as Brazil and India, income is highly skewed and there are large numbers of very poor people in rural communities with limited access to health care. Furthermore, in many countries health care is provided principally by health‐care workers who are not medically or professionally trained, and in others by traditional health practitioners. This may necessitate considerable adaptation of training in, for example, brief advice, to local culture, infrastructure and traditions. A recent international survey of treatment provision found broad agreement with our findings of the affordability of medications globally 13.

This review has identified many important gaps in the tobacco cessation literature. There is little evidence about interventions relating to tobacco use other than cigarette smoking. More evidence is needed on how to deliver effective behavioural support, both in terms of the development of treatment manuals and delivery by individual practitioners. It is also important to identify factors that influence medication effectiveness in real‐world settings, including adherence to the treatment regimen, to assess the potential for internet‐based interventions and how to make interventions more attractive to tobacco users. There are now more than 250 smartphone applications that claim to aid smoking cessation, but these have not yet been evaluated adequately 82. Research is also needed to investigate how to implement brief advice in primary and secondary health‐care in high‐ as well as middle‐ and low‐income countries. Research is needed into the potential for electronic nicotine delivery systems and in methods to improve the reach and/or effectiveness of nicotine replacement therapies. A key priority is how best to motivate and support practitioners and adapt health‐care delivery systems to integrate brief advice into health‐care systems, as recommended by the FCTC Article 14 guidelines.

There is a significant gap in the literature on the effectiveness of combining different interventions. We have used an additive model for the combination of behavioural support and medication, but it is not clear whether this is correct. It could have been anything from multiplicative to partially additive. This is an important area for future research.

Our current understanding of affordability of tobacco cessation interventions could benefit hugely from more advanced economic modelling when better data become available. Until such time, we believe that our customizable spreadsheet will provide local decision‐makers with a practical, evidence‐based tool to help select effective, affordable tobacco cessation interventions for their country.

This review has not considered tobacco harm reduction, defined as ‘measures taken to reduce the harm from continued use of tobacco or tobacco‐derived products’. This typically involves users reducing consumption of cigarettes or switching to less harmful products. For example, the smokeless tobacco, snus, may provide a means of stopping smoking.

A crucial issue with regard to effectiveness, affordability and reach concerns the logistics of delivering interventions and the effects of the setting on these parameters. This will vary considerably across countries and regions, and it is likely that considerable local expertise will be required to establish how interventions can be integrated with existing services.

This review has been produced to support country‐specific national guideline development for smoking cessation by serving as a review of the evidence base, thus removing the need for a country to re‐review the evidence, a process that can be costly and time‐consuming, and to provide a starting point for considering what resources to devote to tobacco cessation support, and what the optimum blend of interventions might be. We hope it will be used alongside a detailed analysis at country level, by all relevant stakeholders working together, to determine cessation priorities within a country.

## Declaration of interests

R.W. undertakes research and consultancy for Pfizer, which manufactures varenicline, and J&J, which manufactures nicotine replacement therapy; he is honorary co‐director of the UK's National Centre for Smoking Cessation and Training and is a trustee of the charity, QUIT; his salary is funded by Cancer Research UK; M.R.: none; A.McN.: none; L.S.: none; P.A. has undertaken research and consultancy for the pharmaceutical industry on smoking cessation; J.B.: none; J.S. was formerly a consultant on clinical trial methodology to several manufacturers of smoking cessation medicines, but has no current involvement with commercial organizations; H.M. has received research funding from and provided consultancy to manufacturers of smoking cessation medications; S.P.: none; A.L.‐G.: none; R.B.: none.

## Acknowledgements

M.R. gratefully acknowledges Bloomberg Philanthropies, who funded his work on this review, and on the design and testing of the affordability calculator. We are also grateful to the Society for the Study of Addiction and Cancer Research UK funding this project.

## Supporting information

Appendix S1 Cochrane Reviews healthcare smoking cessation interventions included in this review.
Appendix S2 Cochrane reviews not included in primary effect size calculation and reasons.

Supporting info item

Supporting info item

Supporting info item
