# Supplementary material for: Health‐care interventions to promote and assist tobacco cessation: a review of efficacy, effectiveness and affordability for use in national guideline development
Source: Addiction. 2015 Jul 29;110(9):1388–403. doi: 10.1111/add.12998 (PMC4737108; doi:10.1111/add.12998)
Supplement: Supplementary file 1 — Supporting info item [file ADD-110-1388-s001.pdf]

Appendix 1: Cochrane Reviews healthcare smoking cessation interventions included in this review

Cochrane Review	Effective intervention
Cahill K, Stead LF, Lancaster T. Nicotine receptor partial agonists for smoking cessation. The Cochrane database of systematic reviews. 2012;4:CD006103.	Varenicline<br>Cytisine
Cahill K, Stevens S, Perera R, Lancaster T. Pharmacological interventions for smoking cessation: an overview and network meta-analysis. The Cochrane database of systematic reviews. 2013;5:CD009329.	Nicotine Replacement Therapy<br>Bupropion<br>Varenicline<br>Nortriptyline<br>Cytisine
Civljak M, Stead LF, Hartmann-Boyce J, Sheikh A, Car J. Internet-based interventions for smoking cessation. The Cochrane database of systematic reviews. 2013;7:CD007078.	Internet-based support
Hartmann-Boyce J, Lancaster T, Stead LF. Print-based self-help interventions for smoking cessation. The Cochrane database of systematic reviews. 2014;6:CD001118.	Printed self-help materials
Hughes JR, Stead LF, Hartmann-Boyce J, Cahill K, Lancaster T. Antidepressants for smoking cessation. The Cochrane database of systematic reviews. 2014;1:CD000031.	Bupropion<br>Nortriptyline
Lancaster T, Stead LF. Individual behavioural counselling for smoking cessation. The Cochrane database of systematic reviews. 2005(2):CD001292.	Face-to-face behavioural support
Stead LF, Buitrago D, Preciado N, Sanchez G, Hartmann-Boyce J, Lancaster T. Physician advice for smoking cessation. The Cochrane database of systematic reviews. 2013;5:CD000165.	Brief advice
Stead LF, Hartmann-Boyce J, Perera R, Lancaster T. Telephone counselling for smoking cessation. The Cochrane database of systematic reviews. 2013;8:CD002850.	Telephone-based behavioural support
Stead LF, Lancaster T. Behavioural interventions as adjuncts to pharmacotherapy for smoking cessation. The Cochrane database of systematic reviews. 2012;12:CD009670.	Face-to-face behavioural support
Stead LF, Lancaster T. Combined pharmacotherapy and behavioural interventions for smoking cessation. The Cochrane database of systematic reviews. 2012;10:CD008286.	Face-to-face behavioural support
Stead LF, Lancaster T. Group behaviour therapy programmes for smoking cessation. The Cochrane database of systematic reviews. 2005(2):CD001007.	Face-to-face behavioural support
Stead LF, Perera R, Bullen C, Mant D, Hartmann-Boyce J, Cahill K, et al. Nicotine replacement therapy for smoking cessation. The Cochrane database of systematic reviews. 2012;11:CD000146.	Nicotine replacement therapy

Whittaker R, McRobbie H, Bullen C, Borland R, Rodgers A, Gu Y. Mobile phone-based interventions for smoking cessation. The Cochrane database of systematic reviews. 2012;11:CD006611.	Text messaging support

For Review Only
