# Supplementary material for: Health‐care interventions to promote and assist tobacco cessation: a review of efficacy, effectiveness and affordability for use in national guideline development
Source: Addiction. 2015 Jul 29;110(9):1388–403. doi: 10.1111/add.12998 (PMC4737108; doi:10.1111/add.12998)
Supplement: Supplementary file 2 — Supporting info item [file ADD-110-1388-s002.pdf]

Appendix 2: Cochrane reviews not included in primary effect size calculation and reasons[1]

Bala MM, Strzeszynski L, Topor-Madry R, Cahill K. Mass media interventions for smoking cessation in adults. The Cochrane database of systematic reviews. 2013;6:CD004704.	Not a healthcare intervention
Barnes J, Dong CY, McRobbie H, Walker N, Mehta M, Stead LF. Hypnotherapy for smoking cessation. The Cochrane database of systematic reviews. 2010(10):CD001008.	Specific component of support
Barth J, Critchley J, Bengel J. Psychosocial interventions for smoking cessation in patients with coronary heart disease. The Cochrane database of systematic reviews. 2008(1):CD006886.	Specific population
Baxi R, Sharma M, Roseby R, Polnay A, Priest N, Waters E, et al. Family and carer smoking control programmes for reducing children's exposure to environmental tobacco smoke. The Cochrane database of systematic reviews. 2014;3:CD001746.	Specific component of support
Bize R, Burnand B, Mueller Y, Rege-Walther M, Camain JY, Cornuz J. Biomedical risk assessment as an aid for smoking cessation. The Cochrane database of systematic reviews. 2012;12:CD004705.	Specific component of support
Boyle R, Solberg L, Fiore M. Use of electronic health records to support smoking cessation. The Cochrane database of systematic reviews. 2011(12):CD008743.	Infrastructure rather than direct intervention
Brinn MP, Carson KV, Esterman AJ, Chang AB, Smith BJ. Mass media interventions for preventing smoking in young people. The Cochrane database of systematic reviews. 2010(11):CD001006.	Not a healthcare intervention
Cahill K, Lancaster T, Green N. Stage-based interventions for smoking cessation. The Cochrane database of systematic reviews. 2010(11):CD004492.	Specific component of support
Cahill K, Lancaster T. Workplace interventions for smoking cessation. The Cochrane database of systematic reviews. 2014;2:CD003440.	Specific setting
Cahill K, Perera R. Competitions and incentives for smoking cessation. The Cochrane database of systematic reviews. 2011(4):CD004307.	Not a healthcare intervention
Cahill K, Ussher MH. Cannabinoid type 1 receptor antagonists for smoking cessation. The Cochrane database of systematic reviews. 2011(3):CD005353.	Not clearly effective[2]
Callinan JE, Clarke A, Doherty K, Kelleher C. Legislative smoking bans for reducing secondhand smoke exposure, smoking prevalence and tobacco consumption. The Cochrane database of systematic	Not a healthcare intervention

reviews. 2010(4):CD005992.	
Carson KV, Brinn MP, Labiszewski NA, Esterman AJ, Chang AB, Smith BJ. Community interventions for preventing smoking in young people. The Cochrane database of systematic reviews. 2011(7):CD001291.	Not a healthcare intervention
Carson KV, Brinn MP, Peters M, Veale A, Esterman AJ, Smith BJ. Interventions for smoking cessation in Indigenous populations. The Cochrane database of systematic reviews. 2012;1:CD009046.	Specific population
Carson KV, Verbiest ME, Crone MR, Brinn MP, Esterman AJ, Assendelft WJ, et al. Training health professionals in smoking cessation. The Cochrane database of systematic reviews. 2012;5:CD000214.	Infrastructure rather than direct intervention
Chamberlain C, O'Mara-Eves A, Oliver S, Caird JR, Perlen SM, Eades SJ, et al. Psychosocial interventions for supporting women to stop smoking in pregnancy. The Cochrane database of systematic reviews. 2013;10:CD001055.	Specific population
Coleman T, Chamberlain C, Davey MA, Cooper SE, Leonardi-Bee J. Pharmacological interventions for promoting smoking cessation during pregnancy. The Cochrane database of systematic reviews. 2012;9:CD010078.	Specific population
Coppo A, Galanti MR, Giordano L, Buscemi D, Bremberg S, Faggiano F. School policies for preventing smoking among young people. The Cochrane database of systematic reviews. 2014;10:CD009990.	Not a healthcare intervention
Critchley J, Capewell S. Smoking cessation for the secondary prevention of coronary heart disease. The Cochrane database of systematic reviews. 2004(1):CD003041.	Specific population
Critchley JA, Capewell S. WITHDRAWN: Smoking cessation for the secondary prevention of coronary heart disease. The Cochrane database of systematic reviews. 2012;2:CD003041.	Specific population
David SP, Lancaster T, Stead LF, Evins AE, Prochaska JJ. Opioid antagonists for smoking cessation. The Cochrane database of systematic reviews. 2013;6:CD003086.	Not clearly effective[2]
Gourlay SG, Stead LF, Benowitz NL. Clonidine for smoking cessation. The Cochrane database of systematic reviews. 2004(3):CD000058.	Not feasible because of side effects
Hajek P, Stead LF, West R, Jarvis M, Hartmann-Boyce J, Lancaster T. Relapse prevention interventions for smoking cessation. The Cochrane database of systematic reviews. 2013;8:CD003999.	Specific component of support

For Review Only

Hajek P, Stead LF. Aversive smoking for smoking cessation. The Cochrane database of systematic reviews. 2004(3):CD000546.	Specific component of support
Hartmann-Boyce J, Cahill K, Hatsukami D, Cornuz J. Nicotine vaccines for smoking cessation. The Cochrane database of systematic reviews. 2012;8:CD007072.	Not clearly effective[2]
Hughes JR, Stead LF, Lancaster T. Anxiolytics for smoking cessation. The Cochrane database of systematic reviews. 2000(4):CD002849.	Not clearly effective[2]
Johnston V, Liberato S, Thomas D. Incentives for preventing smoking in children and adolescents. The Cochrane database of systematic reviews. 2012;10:CD008645.	Not a healthcare intervention
Lai DT, Cahill K, Qin Y, Tang JL. Motivational interviewing for smoking cessation. The Cochrane database of systematic reviews. 2010(1):CD006936.	Specific component of support
Lancaster T, Stead LF. Mecamylamine (a nicotine antagonist) for smoking cessation. The Cochrane database of systematic reviews. 2000(2):CD001009.	Not clearly effective[2]
Lancaster T, Stead LF. Silver acetate for smoking cessation. The Cochrane database of systematic reviews. 2012;9:CD000191.	Not clearly effective[2]
Lovato C, Watts A, Stead LF. Impact of tobacco advertising and promotion on increasing adolescent smoking behaviours. The Cochrane database of systematic reviews. 2011(10):CD003439.	Not a healthcare intervention
Lumley J, Chamberlain C, Dowswell T, Oliver S, Oakley L, Watson L. Interventions for promoting smoking cessation during pregnancy. The Cochrane database of systematic reviews. 2009(3):CD001055.	Specific population
Maziak W, Ward KD, Eissenberg T. Interventions for waterpipe smoking cessation. The Cochrane database of systematic reviews. 2007(4):CD005549.	Not clearly effective[2]
Park EW, Tudiver FG, Campbell T. Enhancing partner support to improve smoking cessation. The Cochrane database of systematic reviews. 2012;7:CD002928.	Specific component of support
Rice VH, Hartmann-Boyce J, Stead LF. Nursing interventions for smoking cessation. The Cochrane database of systematic reviews. 2013;8:CD001188.	Specific health professional group
Rigotti NA, Clair C, Munafo MR, Stead LF. Interventions for smoking cessation in hospitalised patients. The Cochrane database of systematic reviews. 2012;5:CD001837.	Specific population
Sinclair HK, Bond CM, Stead LF. Community pharmacy personnel interventions for smoking cessation. The Cochrane database of systematic	Specific health professional group

For Review Only

reviews. 2004(1):CD003698.	
Stead LF, Hughes JR. Lobeline for smoking cessation. The Cochrane database of systematic reviews. 2012;2:CD000124.	Not clearly effective[2]
Stead LF, Lancaster T. Nicobrevin for smoking cessation. The Cochrane database of systematic reviews. 2006(2):CD005990.	Not clearly effective[2]
Thomas RE, McLellan J, Perera R. School-based programmes for preventing smoking. The Cochrane database of systematic reviews. 2013;4:CD001293.	Not a healthcare intervention
Thomsen T, Villebro N, Moller AM. Interventions for preoperative smoking cessation. The Cochrane database of systematic reviews. 2014;3:CD002294.	Specific population
Tsoi DT, Porwal M, Webster AC. Interventions for smoking cessation and reduction in individuals with schizophrenia. The Cochrane database of systematic reviews. 2013;2:CD007253.	Specific population
Ussher MH, Taylor AH, Faulkner GE. Exercise interventions for smoking cessation. The Cochrane database of systematic reviews. 2014;8:CD002295.	Specific component of support
van der Meer RM, Wagena EJ, Ostelo RW, Jacobs JE, van Schayck CP. Smoking cessation for chronic obstructive pulmonary disease. The Cochrane database of systematic reviews. 2003(2):CD002999.	Specific population
van der Meer RM, Willemsen MC, Smit F, Cuijpers P. Smoking cessation interventions for smokers with current or past depression. The Cochrane database of systematic reviews. 2013;8:CD006102.	Specific population
White AR, Rampes H, Liu JP, Stead LF, Campbell J. Acupuncture and related interventions for smoking cessation. The Cochrane database of systematic reviews. 2014;1:CD000009.	Not clearly effective[2]

[1]Some of these reviews are referred to in the paper to qualify statements about effect size estimates
[2]These are categories of intervention not found to be effective rather than reviews of included interventions where the review failed to show an effect

For Review Only
